# Assessing the Role of Dark Sweet Cherry (*Prunus avium* L.) Consumption on Cognitive Function, Neuropeptides, and Circadian Rhythm in Obesity: Results from a Randomized Controlled Trial

**DOI:** 10.3390/nu17050784

**Published:** 2025-02-24

**Authors:** Shirley Arbizu, Susanne U. Mertens-Talcott, Stephen Talcott, Aaron Riviere, Steven E. Riechman, Giuliana D. Noratto

**Affiliations:** 1 Department of Food Science and Technology, Texas A&M University, College Station, TX 77843, USA; 2 Department of Health and Kinesiology, Texas A&M University, College Station, TX 77843, USA; 3 Department of Nutrition, Texas A&M University, College Station, TX 77843, USA

**Keywords:** dark sweet cherries, cognition, obesity, human clinical study, neuropeptides, circadian rhythm

## Abstract

**Background/Objectives:** Obesity is linked to a higher risk of cognitive impairment. The objective of this single blind randomized trial was to evaluate the impact of dark sweet cherry (DSC) intake on cognitive function in obese adults. **Methods:** Participants (body mass index (BMI): 30–40 kg/m^2^, >18 years, without chronic diseases and/or antibiotic use) consumed 200 mL of DSC drink with 3 g of cherry powder (*n* = 19) or an isocaloric placebo drink (*n* = 21) twice daily for 30 days. Cognitive function was assessed at Day 1 (D1) and Day 30 (D30) using standardized cognitive tests and the NeuroTracker (NT) 3D training program. Blood biomarkers related to cognitive health (neurotensin, substance p, and oxytocin) and circadian rhythm (melatonin and cortisol) were assessed at D1 and D30 using a Luminex multiplex bead-based immunoassay. **Results:** DSC supplementation significantly improved working memory and concentration, as indicated by higher scores in the digit span forward (DSF, *p* = 0.006) and backward (DSB, *p* = 0.01) tests. However, processing speed, sustained attention, and visual spatial skills, assessed through the trail making (TMT) and digit symbol substitution (DSST) tests, as well as visual cognitive performance (VCP) evaluated by the NT program, showed no significant differences between groups. Neurotensin, associated with cognitive deficits, increased in both cherry and placebo groups but was significant only in the placebo group (*p* = 0.007). Similarly, melatonin increased in both groups, reaching significance only in the placebo group (*p* = 0.02), and it correlated positively with IFNγ, suggesting a compensatory response to inflammation. **Conclusions:** These findings suggest DSC supplementation may enhance specific cognitive functions in obese adults. Further clinical trials are needed to confirm these results.

## 1. Introduction

The prevalence of obesity has steadily increased in the U.S. since 1999, and it is expected to affect nearly 50% of the adult population by 2030 [1]. Obesity is a significant public health issue as it is a major risk factor for various illnesses and is associated with poorer outcomes for chronic diseases [1]. Additionally, obesity has been associated with impaired cognitive performance, cognitive decline, and neurodegenerative disorders [2]. Research conducted previously revealed that obese patients exhibited poorer performance in tasks assessing overall cognitive function, particularly in attention and memory [3]. Similarly, deficits in executive function (attention, processing speed, decision making, working memory, concept formation, and problem solving) have been reported in obese subjects [2,3].

Although the mechanisms underlying the relationship between obesity and cognitive dysfunction remain unclear, evidence indicates that inflammation caused by obesity might play a significant role in the development of cognitive impairment [4]. More specifically, the development of low-chronic inflammation in obese individuals may eventually spread into the central nervous system, leading to negative cognitive outcomes [4,5].

Obesity-induced inflammatory processes might contribute to cognitive and behavioral decline by altering genes related to learning and memory, such as neuropeptides [5]. These small proteinaceous peptides are implicated in the dysregulation underlying obesity-associated cognitive dysfunction [6]. Thus, targeting neuropeptides such as neurotensin, substance p and oxytocin might be a beneficial strategy to address cognitive deficits associated under obesity conditions [7]. Neurotensin modulates a range of diverse biological activities including learning and memory processes [6], and it has been positively correlated with perceived stress, anxiety, depression and eating disorders in female obese patients [8]. Substance P is a neuropeptide that influences many physiological processes, such as inflammation, intestinal motility, mucosal secretion and epithelial ion transmission [9]. Moreover, it acts as both a neurotransmitter and neuromodulator in the central nervous system, regulating a variety of behavioral functions, including general and motor activity, pain, food and water intake, anxiety, and memory consolidation [10]. Oxytocin is another neuropeptide that affects cognitive, emotional, and behavioral processes, including food intake and eating disorders [11], and its serum levels have been found to be reduced in type 2 diabetes and obese subjects [12].

Obesity has also been linked to the dysregulation of circadian rhythm. As such, clinical studies have reported the disturbance of hormones that regulate the circadian rhythm such as melatonin [13] and cortisol [14] in obese subjects. Circadian rhythms are internally driven cycles that influence complex cognitive tasks such as selective attention and executive function [15]. In addition, circadian rhythm disruption is considered a risk factor for obesity development.

Polyphenol-rich foods have received particular attention as numerous studies have investigated whether their consumption is associated with cognitive-enhancing effects and neuroprotective benefits [16]. Cherries contain polyphenols such as phenolic acids (i.e., hydroxycinnamic acids) and flavonoids (i.e., anthocyanins), showing high antioxidant and anti-inflammatory potential [17]. These compounds exert neuroprotective effects via multiple mechanisms, including antioxidant activity, anti-inflammatory properties, and the modulation of neuronal signaling pathways. Anthocyanins (ACNs) have been shown to enhance cognitive function by increasing cerebral blood flow, reducing oxidative stress, and inhibiting neuroinflammation [18,19]. ACNs also promote synaptic plasticity and modulate signaling pathways, such as the cAMP response element-binding protein (CREB) pathway, which is critical for memory formation and learning [18,19]. ACNs might also improve cognitive performance by crossing the blood–brain barrier (BBB) and acting as antioxidants to neutralize reactive oxygen species (ROS) [18,19]. Phenolic acids also play a role in neuroprotection by reducing neuroinflammation and oxidative damage [20]. Moreover, phenolic acids may prevent the aggregation of proteins linked to the development of various neurodegenerative diseases associated with cognitive decline, such as Alzheimer’s disease, Parkinson’s disease, and multiple system atrophy [20].

Even though sweet and tart cherries are good sources of polyphenols, tart cherries are rarely eaten as fresh fruit due to their sour taste. In contrast to sweet cherries, tart cherries are commonly processed into products involving heat, which can degrade polyphenols. Still, tart cherries have been credited with the potential to enhance cognitive function and exert neuroprotective effects. For example, the daily supplementation of a diet containing 2% freeze-dried tart cherry powder improved working memory in Fischer 344 aged rats [21]. Likewise, visual sustained attention and subjective memory were significantly improved in older adults after 12-week consumption of 480 mL of tart cherry juice [22]. Previous *in vitro* studies using neuronal cell models have highlighted the potential of sweet cherries in supporting cognitive function and neuroprotection. For example, Matias et al. [23] reported that a phenolic-rich extract from Portuguese Saco sweet cherry culls exhibited strong antioxidant activity in SK-N-MC neuronal cells exposed to oxidative stress. Similarly, Antognoni et al. [24] demonstrated the neuroprotective potential of new sweet cherry cultivars in neuron-like SH-SY5Y cells as these cultivars protected against oxidative stress and upregulated brain-derived neurotrophic factors (BDNF), reinforcing their potential role in preventing neurodegeneration. These findings suggest that sweet cherries might mitigate oxidative stress-related disorders and exert a neuroprotective effect, potentially supporting cognitive function. To our knowledge, only one study investigated the daily consumption of sweet Bing cherry juice (200 mL) over 12 weeks in participants with aging-associated cognitive decline [25]. In this study, the cherry juice intake improved verbal fluency, short-term memory, and long-term memory in older adults with mild-to moderate dementia [25]. Therefore, the influence of dark sweet cherries (DSCs) in obesity-related cognitive dysfunction remains to be investigated. Furthermore, considering that obesity-induced inflammation can disrupt circadian regulation and neuropeptide signaling, leading to impairments in cognitive function, the objective of this study was to evaluate the influence of daily DSC drink consumption for 30 days on cognitive performance, neuropeptides, and circadian rhythm biomarkers in obese adults.

## 2. Materials and Methods

### 2.1. Study Design and Participant Eligibility

**Figure 1:**
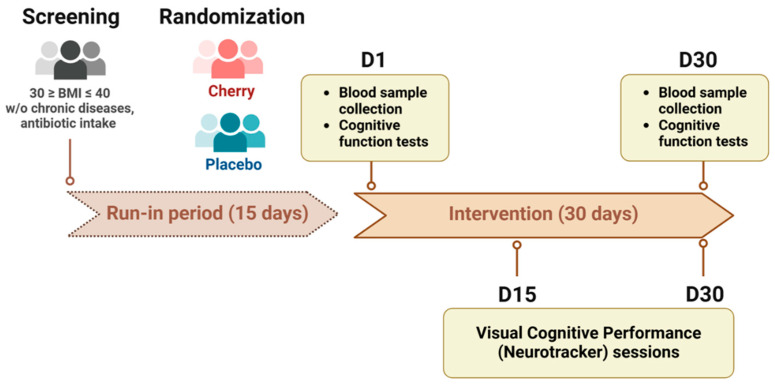
Schematic representation of study protocol. D1: Day 1, D15: Day 15 and D30: Day 30.

The study design and participant eligibility were reported in detail previously [26]. This 30-day single-blind randomized controlled trial was conducted at Texas A&M University (College Station, TX). The decision to conduct a 30-day intervention was based on the study’s complexity with participant compliance and potential dropout rates being critical considerations. Participants were exclusively recruited from the A&M campus, allowing for controlled enrollment and enhanced monitoring, which ensured better compliance with the intervention. Adults aged 18 years and older were recruited between January 2020 and August 2021 based on the following inclusion criteria: body mass index (BMI) ≥30 and ≤40 and no history of chronic diseases or intestinal disorders [26,27]. Participants were excluded if they had any of the following conditions within the previous 6 months: acute cardiac event, stroke, cancer, hepatitis (B or C), HIV, liver or renal dysfunction, macular degeneration, glaucoma, retinitis pigmentosa, optic neuropathy, retinal vascular occlusions, strabismus, autoimmune disorders related to visual health, history of dizziness/fainting during and after blood draws, gluten sensitivity or celiac disease. Antibiotic use, excessive alcohol consumption, smoking, berry allergies or sensitivities, pregnancy or lactation were also considered exclusion criteria [26,27]. After completing a 2-week run-in period, during which participants were instructed to avoid polyphenol-rich foods, an adaptive randomization method was implemented to balance key prognostic factors (BMI, gender, and age) across treatment groups and ensure sample representativeness, minimizing selection bias. After the 2-week run-in period, participants were scheduled for study visits: Day 1 (D1), Day 15 during the intervention (D15), and Day 30 at the end of the study (D30). A schematic representation of the study schedule is presented in Figure 1. This study was approved by the Institutional Review Board (IRB2019-0597F) on 13 November 2019 at Texas A&M University and registered at clinicaltrials.gov as NCT05586386 on 19 October 2022.

### 2.2. DSC Supplementation and Dietary Assessment

On D1, participants received 50 mL of DSC concentrated juice which was kindly provided by FruitSmart^®^ (Grandview, WA, USA) and supplemented with 3 g of DSC powder kindly provided by Anderson Advance Ingredients (Irvine, CA, USA) with instructions to reconstitute with water up to 200 mL (~8 oz). The placebo concentrated drink was prepared by study personnel according to good manufacturing practices in the Department of Food Science and Technology at Texas A&M University. The nutritional and physicochemical characteristics of the DSC concentrated juice, DSC powder, reconstituted DSC drink, and placebo concentrated drink formulation have been previously detailed and can be accessed at https://www.mdpi.com/article/10.3390/nu15030681/s1 (accessed on 29 January 2023). The reconstituted DSC drink contained bioactive compounds from both DSC juice and DSC powder, providing 0.11 g of fiber, 439.6 mg of total phenolics (as gallic acid equivalents), and 70.21 mg of cyanidin 3-glucoside anthocyanins [26,27]. Participants were instructed to drink the reconstituted DSC or placebo beverage twice a day for 30 days and to maintain their usual physical activity and diet. Additionally, participants were provided with both verbal and written instructions to limit their consumption of foods high in polyphenols and to avoid berry intake during the study. Participants were instructed to track their daily food and beverage intake over 15 days during the 30-day intervention using MyFitnessPal (https://www.myfitnesspal.com) to evaluate the impact of their nutritional habits on the study outcomes [26]. Nutritional intake, including calories, carbohydrates, fat, protein, fiber, and cholesterol, was used to estimate Healthy Eating Index (HEI) scores, which reflect how closely a diet aligns with the key recommendations of the Dietary Guidelines for Americans. A detailed description of the data collection and analysis methods has been previously published [26].

### 2.3. Cognitive Performance

Cognitive performance was assessed through the evaluation of executive function skills and visual cognitive performance.

(a) Executive Function Skills: The trail making test (TMT), digit span (DS) and digit symbol substitution test (DSST) were used to evaluate executive function skills at D1 and D30.
  - TMT: Executive attention and visual–spatial functions (visual search, scanning and mental flexibility) were assessed by trail making tests A (TMT-A) and B (TMT-B) [28]. In TMT-A, participants were asked to draw lines to connect numbered circles (1–25) in ascending order. In TMT-B, participants were asked to draw lines to connect circles in an ascending pattern but with the added task of alternating between the numbers (1–13) and letters (A–L). Participants were instructed to complete TMT-A and -B as fast as possible without lifting their pen/pencil from the paper, and the time in which each test was completed was recorded [28]. The results for both TMT-A and TMT-B were expressed as the time in seconds taken to complete the task; thus, higher scores indicated greater cognitive impairment.
  - DS: Working memory, cognitive flexibility and concentration skills were evaluated by the digit span forward (DSF) and backward (DSB) tests [29]. In the DSF test, participants were asked to repeat a set of numbers in the same order as read by the study coordinator, while the DSB test required participants to repeat numbers in the reverse order of that given. Scores were given according to the set of numbers that were repeated correctly. For both DSF and DSB, a higher score indicates better ability to manage and process information in short-term and working memory tasks.
  - DSST: Processing speed, sustained attention, and visual perceptual functions (scanning and basic manual dexterity) were evaluated with DSST [30]. In this test, participants were tasked to associate a collection of symbols with numerical digits within a 90-second timeframe, and their score was determined by the quantity of accurately matched symbols completed within this time limit. In this test, a higher score indicates that an individual processes information quickly and accurately.
(b) Visual Cognitive Performance (VCP): VCP refers to the ability of the brain to interpret and process visual information to perform cognitive tasks effectively [31]. VCP was evaluated during the last 15 days (D15–D30) of the intervention using the NeuroTracker™ CORE program (NT) (CogniSens Athletic Inc., Montreal, QC, Canada). The NT is a computerized 3-dimensional multiple object tracking training (3D-MOT) that evaluates cognitive tasks such as attention, short-term memory, working memory, problem solving, decision making, and information processing speed [32,33]. Participants visited our facility 10× over the last 15 days of the intervention and alternated between 1 or 2 NT training sessions at each visit to complete a total of 15 sessions. During each session, participants were instructed to track the spatial location of 4 target spheres among 4 spheres identical in shape and color. The spheres moved within a 3D virtual space, colliding with one another or the edges of the screen and changing direction, as well as passing in front of or behind one another. The sessions were based on a staircase procedure in which an algorithm shifts the speed of the target spheres in response to the participant’s performance [33]. When participants could identify the target spheres after 6 s of movement, the speed increased by 0.05 log; otherwise, with each incorrect response, the speed decreased by 0.05 log [33]. Participants performed 20 trials within a single NT training session obtaining a speed threshold, which is the level at which participants correctly tracked and selected the correct spheres 50% of the time. Each NT training session was performed in a quiet and dark room. Software was run on a customized Dell computer with NVIDIA GeForce GTX video graphics driver output to a 50″ Sony 3D TV synchronized with active 3D glasses. Each of the 10 visits involved data collection on factors that could affect cognitive performance such as sleep patterns (measured by the Stanford Sleepiness Scale) oxygen saturation (measured with pulseox), hydration levels (evaluated by a validated urine color scale), body weight and composition, blood pressure as well as the timing of exercise and recent fluid consumption [31]. The VCP speed scores were determined throughout the 15 sessions over 10 visits to our facility.

### 2.4. Blood Sample Collection

Participants were instructed to fast for 12 h prior to each study appointment and to consume plenty of water to facilitate blood collection. Blood samples were collected on D1 and D30 in Vacutainer ^®^ SST^TM^ (BD, Franklin Lakes, NJ, USA) to obtain the serum. Blood samples were inverted ~5× to facilitate the clotting process and sited at an ambient temperature for 30 min after collection. Serum was obtained from whole blood left undisturbed at room temperature for 30 min followed by centrifugation at 2000 rpm for 10 min. Serum fractions were aliquoted and stored at −80 °C for analysis of biomarkers related to cognitive dysfunction (neuropeptides) and circadian regulation (cortisol and melatonin).

(a) Analysis of neuropeptides

Serum samples collected on D1 and D30 were used to assess neuropeptide levels using the Milliplex ^®^ MAP Human Neuropeptide Magnetic Bead Panel (EMD Millipore; Billerica, MA, USA) according to the manufacturer’s protocol. Analytes included neurotensin, substance p and oxytocin. Data were analyzed using the Luminex xPonent 3.1 software.

(b) Analysis of circadian biomarkers

Circadian rhythm biomarkers, cortisol and melatonin were quantified in serum samples collected on D1 and D30 using the Human Circadian/Stress Magnetic Bead Panel (EMD Millipore; Billerica, MA, USA) following the manufacturer’s instructions. Analytes included cortisol and melatonin. Data were analyzed using the Luminex xPonent 3.1 software.

### 2.5. Statistical Analysis

Statistical analyses were performed using Graphpad Prism Software (Version 9.3.1, La Jolla, CA, USA). Descriptive statistics were calculated and data are presented as mean with 95% confidence interval (95% CI) unless otherwise indicated. Normality was assessed using the Shapiro–Wilk test, and data not following a normal distribution were log-transformed and noted accordingly. The differences in D1 values between the cherry and placebo groups were assessed using parametric unpaired t-tests. Age, gender, BMI, physical activity, and HEI scores were considered as covariates, but their impact on the dependent variables was found to be non-significant (*p* > 0.05) [26,27].

Data on executive function skills, neuropeptides and circadian rhythm were analyzed by the repeated measures (RM) two-way ANOVA test followed by Sidak’s multiple comparisons. The effect of treatment, day and their interaction were evaluated. In addition, the mean change (Δ) D30-D1 was computed for each treatment and used to examine treatment differences by the unpaired-t test. Data statistically significant due to treatment or day effect were stratified by BMI (low: 30–34, high: 35–40) and gender (male, female).

A mixed-effect analysis compared the effect of DSC intake on VCP which was assessed using the 15-day NT speed threshold. In addition, the speed threshold analysis included (a) evaluating mean performance and (b) comparing the baseline NT sessions (sessions 1–3 or baseline 3) to the final NT sessions (sessions 13–15 or final 3) and (c) comparing Δ (final baseline) values. For all analyses, a *p*-value < 0.05 was considered statistically significant.

Spearman correlation analyses were performed in GraphPad Prism to examine associations between significant changes in cognitive performance, obesity-related biomarkers and gut microbiota composition previously evaluated and published following DSC supplementation [26,27]. Correlations with r > 0.40 and *p* < 0.05 were considered indicative of a moderate association between the variables examined [34].

## 3. Results

### 3.1. Participant Characteristics and Nutritional Assessment

As previously reported, 40 participants successfully completed the study (19 in the cherry group: 11 females and 8 males; and 21 in the placebo group: 14 females and 7 males) [26,27]. Comprehensive data on anthropometric and physiological measurements, nutrient intake, and HEI results have been documented and are available at https://www.mdpi.com/2072-6643/15/3/681 (accessed on 29 January 2023). Briefly, there were no significant differences in anthropometric measurements (body weight, BMI, body fat, and waist circumference) or physiological measurements (heart rate and oxygen saturation) between the cherry and placebo groups after the 30-day intervention [26]. However, DSC supplementation resulted in a reduction in systolic blood pressure (SBP) (*p* = 0.05) and diastolic blood pressure (DBP) (*p* = 0.04) at D30 compared to the placebo group [26]. Analysis of 15-day dietary records revealed no significant differences between the study groups in mean nutrient intake and HEI scores [26].

### 3.2. Cognitive Performance

#### 3.2.1. Executive Function Skills

At D1, the scores for TMT-A and TMT-B were similar between cherry and placebo groups. However, at D30, participants in the cherry and placebo groups showed significantly lower TMT-A scores compared to D1 (Table 1), implying similar improvement in executive attention and visual–spatial function in both groups. Moreover, Δ TMT-A and Δ TMT-B analyses showed no significant differences between groups (Supplementary Table S1). Both TMT-A and TMT-B results for participants in the cherry and placebo groups were within the average range (29 s for TMT-A and 75 s for TMT-B) [35], suggesting that participants did not show impairments in these cognition markers.

**Figure 2:**
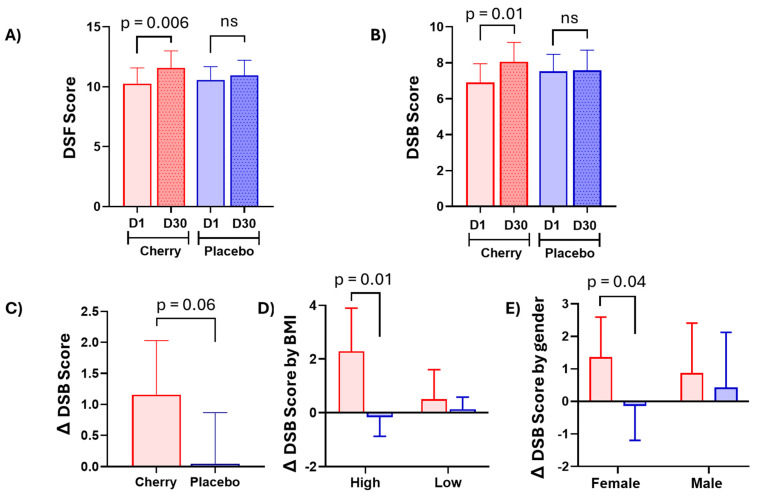
DSC supplementation improved working memory in adults with obesity. (**A**) DSF and (**B**) DBS scores at D1 and 30 in cherry and placebo groups. Values are mean, 95% CI. The difference between treatments was assessed by Sidak’s multiple comparison test (**C**) Δ DSB in cherry and placebo groups. (**D**) Δ DSB scores stratified by BMI and (**E**) gender in cherry and placebo groups. Values are mean, 95% CI. The difference between treatments was assessed by unpaired *t*-tests. Δ = D30-D1. DSF: digit span forward, DSB: digit span backward, ns: not significant.

Working memory and attention skills, assessed by the DSF and DSB tests, revealed that participants in both the cherry and placebo groups had scores that did not suggest cognitive impairment [36]. However, significant changes were detected only in the cherry group, as evidenced by significantly higher DSF (*p* = 0.006) and DSB (*p* = 0.01) scores at D30 vs. D1, while no significant changes were detected in the placebo group (Table 1, Figure 2A,B). Furthermore, Δ DSB in the cherry group was higher than in the placebo group but did not reach significance (*p* = 0.06) due to the high variability among participants (Figure 2C and Supplementary Table S1). Stratification analyses revealed that the statistical differences in Δ DSB were significant for participants with high BMI (*p* = 0.01) and females (*p* = 0.04) (Figure 2D,E, Supplementary Table S2).

Participants in the cherry and placebo groups showed improvements in processing speed, sustained attention, and visual perceptual functions as evidenced by higher DSST scores at D30 vs. D1 at similar extent (Table 1).

#### 3.2.2. Visual Cognitive Performance (VCP)

The mixed effect analysis showed that VCP speed scores, assessed by the NT training system, improved significantly in the cherry and placebo groups (Supplementary Figure S1A). The final VCP scores improved significantly in the cherry (*p* < 0.0001) and placebo (*p* = 0.0001) groups compared to their respective baseline values, while no significant differences were detected between treatments (Table 2). Similarly, Δ VCP in the cherry group and Δ VCP in the placebo group showed no significant differences between treatments (Table 2). Stratification analyses showed that high BMI and female participants in the cherry group had slightly higher VCP scores (1.54 and 1.47, respectively) compared to those in the placebo group (1.06 and 1.41, respectively) at D30, but the differences among treatments were not significant (Supplementary Figure S1B,C and Supplementary Table S3). The mean values of variables assessed before each NT session showed significant differences in hydration status, as indicated by urine color, between the cherry and placebo groups. A similar outcome was detected for SBP and DBP, which were measured before each NT session (Supplementary Table S4). These variables were used as covariates to analyze the differences between Δ VCP scores in the cherry and placebo groups; however, these differences were deemed non-significant (Table 2).

### 3.3. Neuropeptides and Circadian Rhythm Biomarkers

**Figure 3:**
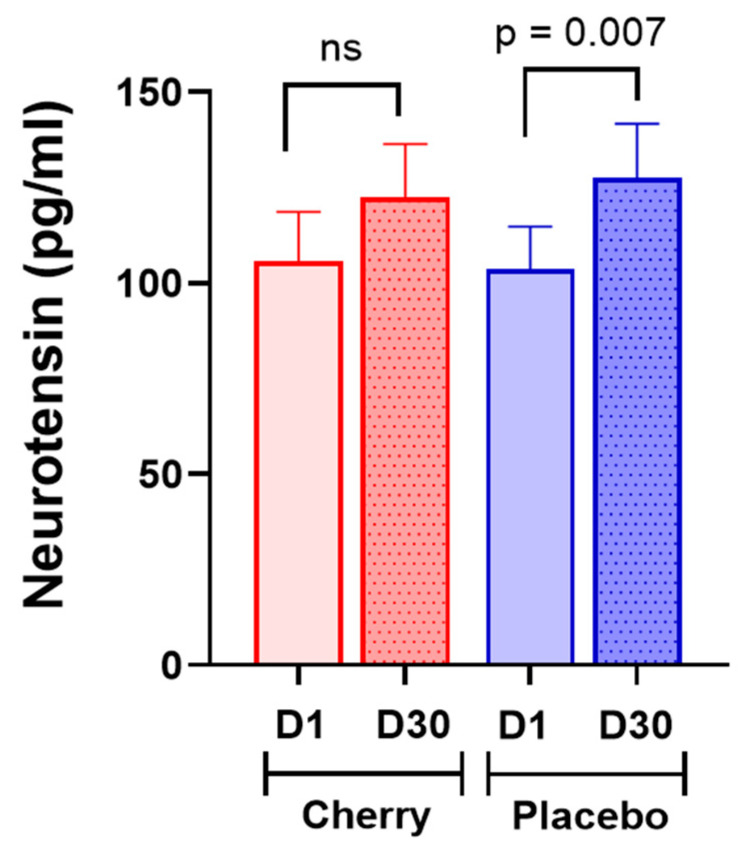
Neurotensin increased significantly in the placebo group. The difference between treatment was assessed by Sidak’s multiple comparison test. Values are mean, 95% CI. Ns: non-significant.

Serum levels of neuropeptides and circadian rhythm biomarkers are presented in Table 3. Neurotensin, a neuropeptide with a multifaceted role in cognitive processes [6], increased significantly in the placebo group at D30 (*p* = 0.007) compared to D1 values (Table 3, Figure 3), while in the cherry group, there was a trend to increase at D30 vs. D1 without reaching significance. However, Δ neurotensin did not differ significantly between treatment groups (Supplementary Table S5). Similarly, substance p and oxytocin showed a trend of increase in both cherry and placebo groups (Table 3), though neither Δ substance p nor Δ oxytocin reached statistical significance between treatments (Supplementary Table S5).

The levels of cortisol decreased in the cherry group, whereas levels in the placebo group showed an opposite trend at D30 vs. D1 values without reaching significance (Table 3). Serum melatonin increased in the cherry and placebo groups; however, only significant changes were detected in the placebo group (*p* = 0.02) (Table 3). Similarly, Δ cortisol and Δ melatonin did not show significant differences between the cherry and placebo groups (Supplementary Table S5).

### 3.4. Correlation Analyses of Significant Cognitive Function Biomarkers Assessed During Intervention

Previously, it was reported that DSC juice supplementation lowered obesity-related biomarkers such as SBP and DBP as well as decreased pro-inflammatory interferon gamma (IFNγ) in obese adults [26]. Similarly, we also reported that DSC intake decreased the abundance of bacteria associated with inflammation such as *Alistipes shahii* and *Bilophila* and prevented the decrease in *Bifidobacterium* genus, which has been suggested to play a role in reducing symptoms of anxiety and depression [27]. Interestingly, results from the Spearman correlation analysis in the placebo group showed that Δ neurotensin was positively correlated with Δ *A. hadrus* (r = 0.43, *p* = 0.04), whereas Δ melatonin showed a significant positive correlation with Δ IFNγ (r = 0.54, *p* = 0.02) (Supplementary Table S6). Such correlations were not found in the cherry group.

## 4. Discussion

To our knowledge, this is the first study investigating the role of DSC supplementation in cognitive function, neuropeptides, and circadian rhythm biomarkers in obese adults. Obesity is associated with cognitive decline and elevated risk of neurodegenerative diseases [2,3]. As such, obese individuals show deficits in learning, memory, and executive function compared to normal-weight subjects [2,3]. The results from this study suggest that DSC supplementation may improve specific cognitive abilities in adults with obesity such as working memory and concentration skills as assessed by the DSF and DSB tests. Interestingly, improvements in the DSB tasks were more apparent in females and high BMI participants. Given the increased risk of cognitive impairments and neurological disorders in individuals with obesity, these findings support the potential of DSC as a promising dietary intervention to improve cognitive health. Moreover, DSC supplementation showed a trend toward improved executive attention and visual–spatial functions evaluated by the TMT-A, but the high variability between subjects prevented it from reaching significance. The findings from our study are in line with a clinical study that reported improvements in verbal fluency as well as short and long-term memory in older adults with mild-to moderate dementia after 12-weeks of sweet Bing cherry juice supplementation (200 mL) [25]. However, our study was conducted over 30 days (~4 weeks) in adults with obesity, and our results suggest that cognitive improvements may occur over a shorter duration in this population. This has important implications for dietary and clinical interventions, as it indicates that the regular consumption of polyphenol-rich foods like DSCs may exert acute effects on cognitive function in individuals at risk of cognitive impairment within a shorter duration.

The dietary intake of polyphenol-rich foods, such as blueberries, blackcurrants, grapes, and strawberries, is associated with enhanced cognitive function and a reduced risk of neurodegenerative diseases in young and older adults [37,38,39,40]. This suggests that polyphenol compounds contribute to preventing cognitive decline across different age groups. DSC is a rich source of polyphenols, including a variety of flavonoids and phenolic acids which contribute to their strong antioxidant properties. The DSC juice used on this study had anthocyanins (ACNs) and flavan-3-ols as the most prevalent (approximately 22% and 29%, respectively) polyphenols, and lower amounts of flavonols and phenolic acids (approximately 8% and 12%, respectively) [41]. These compounds have been associated with cognitive enhancement properties and might play a role in improving working memory and concentration skills in obese adults. For example, ACNs might cross the BBB, allowing them to exert direct effects within the central nervous system. Once in the brain, they neutralize ROS, thereby reducing oxidative stress, which is a key factor in cognitive decline [18,19,42,43]. In addition, ACNs might also inhibit the activation of pro-inflammatory signaling pathways, such as nuclear factor-kappa B (NF-κB), and reduce the production of pro-inflammatory cytokines. This anti-inflammatory action is thought to contribute to the neuroprotective effects of ACNs, such as improved memory, learning, cognitive, and motor functions [44]. Similarly, flavan-3-ols and other classes of flavonoids play a crucial role in supporting cognitive function by mitigating oxidative stress and neuroinflammation [45]. Phenolic acids contribute to cognitive function through multiple neuroprotective mechanisms. They help reduce oxidative stress by lowering pro-oxidant levels, enhancing antioxidant activity, and increasing the production of anti-inflammatory mediators [20], supporting cognitive processes such as learning and memory [20]. However, these beneficial effects appear to be selective on certain cognitive domains. As such, verbal memory is consistently reported as a cognitive domain showing the greatest sensitivity to polyphenols supplementation [16]. This evidence might support the results of this study, as DSC supplementation significantly improved working memory and concentration skills assessed by the DSF and DSB tasks, while other executive function domains (executive attention, processing speed and visual spatial skills) assessed by TMT-A and -B and DSST were partially or not improved. Consequently, the extent to which DSC may provide protective effects on cognitive domains altered during obesity deserves further investigation.

Overall, the mechanism of action behind polyphenols’ cognitive enhancement properties is thought to be exerted by their capacity to mitigate inflammatory markers, resulting in blood pressure-lowering effects [25]. For example, Chai et al. [46] reported lower SBP and C-reactive protein (CRP) levels in older adults after the intake of tart cherry juice for 12 weeks. Interestingly, these results were accompanied by improved visual sustained attention and spatial working memory [22]. Similarly, Kent et al. [25] demonstrated a significant improvement in cognitive abilities and decreased SBP levels in older adults with mild-to moderate dementia after a 12-week intake of sweet cherry juice (200 mL). We previously reported that DSC intake significantly decreased SBP and DBP, which were positively correlated with lower levels of pro-inflammatory IFNγ [26]. However, no direct correlation was found between IFNγ, blood pressure levels and cognitive tests assessed in this study. This suggests that while the anti-inflammatory and blood pressure-lowering effects of DSC might contribute to the observed cognitive benefits, these factors alone do not fully explain the improvements. Other mechanisms, potentially beyond inflammation and blood pressure regulation, may be involved in enhancing cognitive function and should be further investigated.

The lack of effect observed in the TMT-A and -B, DSST and VCP could be attributed to the duration of DSC intervention. Based on previous findings, the cognitive improvement effects of tart and sweet cherries were reached through a minimum of 12 weeks supplementation [22,25], as it has been suggested that polyphenols may take several weeks to accumulate in brain tissues [22]. Thus, it cannot be excluded that interventions longer than 30 days might be required to elicit enhanced cognitive abilities in obese adults.

In addition to polyphenols, DSCs are also a source of dietary fiber (DF), which might also contribute to their cognitive health benefits. DF is fermented in the lower gastrointestinal tract and may enhance cognitive function by promoting a greater microbial diversity to produce a variety of health-promoting metabolites [47]. For example, better performance on memory tasks was reported in healthy older adults after the daily intake of a berry beverage high in ACNs (~795 mg) and fiber (11 gr) for 5 weeks [40]. In this study, the ACN and fiber content were lower (~70 mg/200 mL and 0.11 gr, respectively) [26] and the study duration was shorter (30 days or ~4 weeks), which might explain why executive attention, processing speed and visual spatial skills were not improved.

Research on neurotensin suggests it plays a role in both obesity and cognitive function, often intersecting with neuroinflammatory pathways that can contribute to cognitive deficits [48]. Serum neurotensin increased significantly in the placebo group, while levels in the cherry group tended to increase without attaining significance, suggesting the protective effects of DSC supplementation against cognitive deficits in obese subjects. Interestingly, a positive relationship between Δ neurotensin and Δ *A. hadrus* was detected only in the placebo group. *A. hadrus* is a butyrate-producing bacterium that promotes anti-inflammatory responses and supports gut barrier integrity; however, its presence might exacerbate inflammation under dysbiosis or in combination with other pro-inflammatory factors [27]. We previously reported an unchanged abundance of Δ *A. hadrus* in the placebo group [27], suggesting that increased neurotensin levels might correlate with an unchanged abundance of *A. hadrus*, potentially contributing to cognitive deficits.

Currently, no previous studies have investigated the potential of DSC in the modulation of neurotensin; thus, the comparability of results from this study is limited. However, it was previously suggested that some flavonoids may have the potential to modulate neuropeptides involved in food intake and satiety (e.g., neuropeptide Y) due to their capacity to cross the BBB where they can exert antioxidant and anti-inflammatory effects [49] and promote cognitive health. In this study, the lack of effect observed on substance p and oxytocin could be attributed to the relatively short DSC intervention.

Circadian rhythms are internally driven cycles that influence complex cognitive tasks such as attention, working memory and executive function [15]. The disruption of the circadian system is considered a risk factor for obesity, type 2 diabetes, and metabolic syndrome development. Cortisol, commonly known as the “stress hormone”, plays an essential role in metabolic and immune processes, the diurnal sleep–wake cycle, stress response, and blood pressure regulation [50]. In addition, increased cortisol levels are strongly related to abdominal obesity and to specific mental disorders [51]. In this study, cortisol levels tended to decrease following the DSC intervention, whereas an opposite trend was observed in the placebo group. Stratification analysis further indicated that this trend was more pronounced among women in the cherry group compared to those in the placebo group. However, the difference did not reach significance (*p* = 0.08) due to the high variability among participants. These findings warrant further investigation, as increased levels of cortisol might interfere with cognitive processes and further aggravate mental disorders (stress, anxiety, and depression) associated with obesity [51,52].

Cherries are considered an important source of melatonin, and their daily consumption might increase the endogenous production of melatonin, enhancing antioxidant and anti-inflammatory effects as well as improving sleep quality in adults with metabolic disorders [53,54]. Unexpectedly, melatonin levels increased significantly in the placebo group by D30, while levels in the cherry group remained unchanged. These results may seem controversial because cherries are presented as a good source of exogenous melatonin and as a protective dietary strategy against obesity-associated metabolic disturbances. However, elevated levels of melatonin in placebo participants may be a compensatory mechanism to counteract the higher inflammatory status. Melatonin can inhibit the translocation of NF-κB to the nucleus, preventing the upregulation of inflammatory cytokines, which helps reduce inflammation [55]. Therefore, the upregulation of melatonin observed in the placebo might be due to the endogenous melatonin synthesis to counteract obesity-associated inflammation as evidenced by the strong correlation between Δ melatonin and Δ IFNγ found only in the placebo group (r = 0.54, *p* = 0.02).

Results from this study should be considered exploratory and interpreted with caution because the measurement of melatonin was performed on serum samples collected after ~12 h fasting, and this might represent a limitation in terms of evaluating melatonin status. Melatonin levels should be measured in samples collected around sleep time because melatonin is predominantly secreted at night. However, fasting blood samples were necessary for assessing additional biomarkers, including inflammatory markers, lipid profile, glucose, and lipopolysaccharide-binding protein, as part of our study’s broader objectives, and published previously [26,27]. Given that these measurements required a standardized fasting state, the collection time could not be adjusted specifically for circadian rhythm assessment. The evaluation of urine 6-sulphatoxymelatonin (6-SMT) in urine, which is a metabolite of melatonin, should be performed in further studies, since it is considered an accurate and reliable method to explore the impact of dietary interventions in melatonin profile [56].

The strengths of this study include that participants in the cherry group did not report serious adverse effects, and compliance was 90%, suggesting DSC drink as a safe and healthier alternative to artificially sweetened beverages, since no significant changes in BMI, body weight, fat and blood glucose were detected after the 30-day intervention [26]. Limitations of this study include the timing and scope of physical activity monitoring. We controlled physical activity halfway through the intervention (cherry: *n* = 8, placebo: *n* = 11) and assessed whether it was a significant covariate. Our analysis indicated that physical activity did not significantly impact the results. However, we acknowledge that monitoring halfway through the study does not account for variations in activity levels before or after the monitoring period, which could still influence cognitive outcomes.

A longer DSC intervention lasting up to 12 weeks may be necessary to observe meaningful improvements in various aspects of cognitive function, as indicated by studies involving sweet and tart cherries. Moreover, longer interventions may reveal benefits in more complex cognitive domains, such as executive function, processing speed, and working memory, which may require sustained exposure to bioactive compounds to show measurable improvements.

A longer intervention period could also help reduce practice effects associated with cognitive tasks, which might mask a decline in cognitive performance or overestimate the effects of the intervention [22,25,57]. The exposure to cognitive tests might have increased familiarity with the tasks, leading to learning or memory effects that improved participants’ performance over time. To better account for these practice effects in future studies, it would be useful to include alternative versions of the tasks or introduce longer intervals between assessments. Additionally, counterbalancing the order of task administration could help reduce the impact of repeated testing.

Participants in both the cherry and placebo groups showed no apparent cognitive deficiencies, as their TMT-A TMT-B, DSF and DSB scores within normal ranges [35,58]. This raises the question of whether DSC supplementation could be beneficial for individuals without cognitive impairment. While previous studies have shown that sweet and/or tart cherries can slow or reverse age-related cognitive decline, this is the first to assess cognitive performance in obese adults in response to DSC consumption. Results from this study are promising and provide a platform for future research investigating the role of DSC interventions on populations showing cognitive dysfunction associated with obesity.

## 5. Conclusions

This study showed that daily DSC drink intake might enhance working memory and concentration skills in obese adults. Although DSC consumption did not decrease neurotensin levels, it helped prevent a significant increase observed in the placebo group, suggesting the protective effects of DSC drink intake against cognitive deficits related to learning and memory processes in obese subjects. Increased melatonin levels were significantly correlated with IFNγ in the placebo group, implying a mechanism to counteract inflammation. Further clinical studies with larger sample sizes and longer durations are necessary to confirm these effects and explore the long-term clinical application of DSC supplementation. Finally, this study highlights DSC drink as a potential dietary strategy aimed at promoting cognitive health in obese adults who are under greater risk to develop cognitive impairments and neurological disorders.

## Figures and Tables

**Table 1:** Cognitive function tasks scores before and after intervention in cherry and placebo groups.

Test	Day	Treatment		2-Way ANOVA *p* Values			Sliced by Treatment and/or Day
		Cherry (*n* = 19)	Placebo (*n* = 21)	Treatment	Day	Interaction	
TMT-A	D1	29.2 (24.4, 34.2) ^X^	27.9 (24.8, 31.1) ^X^	*0.75*	*0.0004*	*0.99*	Placebo (*p* = 0.01) ↓ D30 Cherry (*p* = 0.02) ↓ D30
	D30	24.6 (21.2, 28.0) ^Y^	24.3 (20.8, 27.9) ^Y^				
TMT-B	D1	61.2 (50.5, 72.0)	56.5 (48.9, 64.2) *n* = 20	0.44	0.03	0.78	ns
	D30	53.1 (44.1, 62.0)	50.1 (41.7, 58.5) *n* = 20				
DSF	D1	10.2 (8.9, 11.5) ^X^	10.5 (9.4, 11.6)	*0.88*	*0.007*	*0.09*	Cherry (*p* = 0.006) ↑ D30
	D30	11.6 (10.2, 12.9) ^Y^	10.9 (9.6, 12.2)				
DSB	D1	6.9 (5.8, 7.9) ^X^	7.5 (6.7, 8.4)	0.91	0.04	0.06	Cherry (*p* = 0.01) ↑ D30
	D30	8.1 (6.9, 9.1) ^Y^	7.6 (6.5, 8.6)				
DSST	D1	63.5 (58.8, 68.2)	61.5 (55.8, 67.1)	0.63	0.008	0.92	ns
	D30	67.3 (60.2, 74.3)	65.5 (58.7, 72.1)				

Values are mean (95% CI). The impact of treatment, day and the interaction (treatment and day) was assessed by repeated measures 2-way ANOVA, which was followed by Šidák multiple comparison. Statistically significant results (*p* < 0.05) are represented by the letters X and Y (indicating significant differences within treatment). TMT-A: trail making test A, TMT-B: trail making test B, DSF: digit span forward, DSB: digit span backward, DSST: digit symbol substitution test. Italicized *p* values are for log transformed data; untransformed data are presented. ns: not significant. ↑: increase, ↓: decrease. ns: not significant.

**Table 2:** Visual cognitive performance (VCP) speed scores in cherry and placebo groups.

	Cherry (*n* = 19)	Placebo (*n* = 20)	*p* Value
Mean of 15	1.35 (1.18, 1.52)	1.37 (1.16, 1.58)	0.89
Baseline (3)	1.23 (1.04, 1.41)	1.20 (0.97, 1.42)	0.81
Final (3)	1.50 (1.34, 1.65) *	1.44 (1.20, 1.68) *	0.69
Mean change (Δ)	0.26 (0.15, 0.37) ^#^	0.25 (0.14, 0.36) ^#^	0.94

Values are mean, 95% CI. Baseline values are from the first 3 NT sessions, and final values are from the last 3 NT sessions. Mean change in VCP (Δ): Final (3) - baseline (3). (*) Indicates significant difference between baseline and final values, *p* ≤ 0.0001 for cherry and *p* = 0.0001 for placebo. (^#^) Δ values were adjusted for significant urine, SBP and DBP values.

**Table 3:** Blood levels of neuropeptides and circadian biomarkers before and after intervention in cherry and placebo groups.

Variable	Day	Treatment		*p* Values			Sliced by Treatment and/or Day
		Cherry (*n* = 19)	Placebo (*n* = 21)	Treatment	Day	Interaction	
Neuropeptides							
Neurotensin (pg/mL)	D1	105.81 (92.95, 118.67) *n* = 18	103.70 (92.65, 114.75) ^X^	*0.89*	*0.001*	*0.52*	Placebo (*p* = 0.007) ↑ D30
	D30	122.41 (108.5, 136.6) *n* = 18	127.57 (113.48, 141.67) ^Y^				
Substance p (pg/mL)	D1	21.94 (18.26, 25.61) *n* = 18	21.40 (18.13, 24.67)	*0.63*	*0.09*	*0.59*	ns
	D30	28.65 (19.55, 37.76) *n* = 18	27.59 (24.11, 31.07)				
Oxytocin (pg/mL)	D1	96.75 (68.36, 125.15) *n* = 18	97.95 (69.39, 126.51)	0.94	0.06	0.86	ns
	D30	126.11 (95.19, 157.03) *n* = 18	122.64 (95.24, 150.04)				
Circadian Rhythm							
Cortisol (ng/mL)	D1	170.6 (86.4, 254.9) *n* = 18	215.4 (131.299.3)	*0.40*	*0.70*	*0.71*	ns
	D30	167. 4 (74.7, 260.1) *n* = 18	276.2 (113.9, 404.8)				
Melatonin (pg/mL)	D1	3.36 (2.43, 4.29) *n* = 18	3.37 (2.30, 4.45) ^X^ *n* = 17	*0.34*	*0.008*	*0.29*	Placebo (*p* = 0.02) ↑ D30
	D30	4.97 (3.05, 6.89) *n* = 18	14.41 (4.01, 24.80) ^Y^ *n* = 17				

Values are mean (95% CI). The impact of treatment, day and the interaction (treatment and day) was assessed by repeated measures 2-way ANOVA, which was followed by Šidák multiple comparison. Statistically significant results (*p* < 0.05) are represented by the letters X and Y (indicating significant differences within treatment). Italicized *p* values are for log transformed data; untransformed data are presented. ↑: increase.

## Data Availability

The original contributions presented in the study are included in the article/Supplementary Materials, further inquiries can be directed to the corresponding authors.

## Supplementary Materials

The following supporting information can be downloaded at https://www.mdpi.com/article/10.3390/nu17050784/s1, Figure S1. Participants in the cherry group showed a trend for higher Visual cognitive performance (VCP) speed scores. VCP scores in (A) cherry and placebo groups, (B) VCP scores in cherry and placebo groups stratified by high BMI (35–40), and (C) gender: female participants. VCP was assessed by the Neurotracker 3D training system. Values are mean ± SEM. Table S1. Analysis of Δ TMT-A, Δ TMT-B, Δ DSF, Δ DSB and Δ DSST between cherry and placebo groups. Table S2. Variation of Δ DSB scores according to BMI and gender in cherry and placebo groups. Table S3. Stratification analysis of VCP scores by BMI and gender: female between cherry and placebo groups. Table S4. Variables assessed before visual cognitive performance using the Neurotracker training system in cherry and placebo groups. Table S5. Analysis of Δ Neuropeptides and Δ Circadian rhythm biomarkers in cherry and placebo groups. Table S6. Spearman correlation analysis in cherry and placebo groups.
