# Supplementary material for: Assessing the Role of Dark Sweet Cherry (Prunus avium L.) Consumption on Cognitive Function, Neuropeptides, and Circadian Rhythm in Obesity: Results from a Randomized Controlled Trial
Source: Nutrients. 2025 Feb 24;17(5):784. doi: 10.3390/nu17050784 (PMC11901987; doi:10.3390/nu17050784)
Supplement: Supplementary file 1 [file nutrients-17-00784-s001.zip › Supplementary Tables.pdf]

Table S1. Analysis of Δ TMT-A, Δ TMT-B, Δ DSF, Δ DSB and Δ DSST between cherry and placebo groups.

Test	Treatment		Difference (Cherry-Placebo)	
	**Cherry (n=19)**	**Placebo (n=21)**	**Mean, 95% CI**	**p-value**
**Δ TMT-A**	-4.57 (-8.20, -0.95)	-3.57 (-7.01, -0.12)	-1.00 (-6.00, 3.99)	*0.99*
**Δ TMT-B**	-8.15 (-17.81, 1.49)	-6.35 (-15.76, 3.06) n = 20	-1.80 (-15.29, 11.67)	0.78
**Δ DSF**	1.31 (0.36, 2.26)	0.38 (-0.52, 1.28)	0.93 (-0.37, 2.24)	*0.15*
**Δ DSB**	1.15 (0.32, 1.99)	0.04 (-0.74, 0.84)	1.11 (-0.04, 2.26)	0.06
**Δ DSST**	3.73 (-0.31, 7.79)	4.00 (0.14, 7.85)	-0.26 (-5.86, 5.33)	0.92

Values are mean (95% CI). Differences between cherry and placebo groups were analyzed by unpaired t test. TMT-A: trail making test A, TMT-B: trail making test B, DSF: digit span forward, DSB: digit span backward, DSST: digit symbol substitution test. Δ: D30-D1. Italicized p values are for log transformed data; untransformed data are presented.

Table S2. Variation of Δ DSB scores according to BMI and gender in cherry and placebo groups.

Variable	Treatment		Difference (Cherry-Placebo)	
	**Cherry**	**Placebo**	**Mean, 95% CI**	**p-value**
Gender				
Female	1.36 (0.24, 2.48) n=7	-0.14 (-1.13, 0.85) n=6	1.50 (0.01, 3.00)	0.04
Male	0.87 (-0.43, 2.18) n=12	0.43 (-0.97, 1.83) n=15	0.44 (-1.47, 2.37)	0.64
BMI				
High	2.28 (0.95, 3.62) n=7	-0.16 (-1.60, 1.27) n=6	2.45 (0.48, 4.41)	0.01
Low	0.50 (-0.51, 1.51) n=12	0.13 (-0.77, 1.04) n=15	0.36 (-1.00, 1.73)	0.59

Values are mean (95% CI). Differences between cherry and placebo groups were analyzed by unpaired t test. Δ: D30-D1.

Table S3. Stratification analysis of VCP scores by BMI and gender: female between cherry and placebo groups

	Cherry	Placebo	p-value
High BMI			
Baseline (3)	1.22 (0.97, 1.47)<br>n = 7	0.85 (0.74, 0.9<br>n = 6	0.10
Final (3)	1.54 (1.28, 1.80)<br>n = 7	1.06 (0.92, 1.20)<br>n = 6	0.10
Mean change (Δ)	0.31 (0.18, 0.45)	0.20 (0.13, 0.27)	0.07
Female			
Baseline (3)	1.17 (0.77, 1.57)<br>n = 11	1.20 (0.97, 1.43)<br>n = 12	0.99
Final (3)	1.47 (1.24, 1.69)<br>n = 11	1.41 (1.29, 1.53)<br>n = 12	0.60
Mean change (Δ)	0.29 (0.08, 0.50)	0.21 (0.03, 0.45)	0.23

Values are mean, 95% CI. Differences between treatments were assessed by the Mann-Whitney test.
Mean change (Δ) = Final (3) – baseline (3)

Table S4. Variables assessed before visual cognitive performance using the Neurotracker training system in cherry and placebo groups.

Variable	Treatment		Difference (Cherry-Placebo)	
	Cherry	Placebo	Mean, 95% CI	p-value
Urine color (AU)	2.72 (2.64, 2.80)	2.21 (2.09, 2.33)	0.50 (0.37, 0.64)	<0.0001
Sleep (hr.)	6.75 (6.49, 7.02)	6.81 (6.68, 6.94)	-0.05 (-0.32, 0.22)	0.68
Standford Sleepiness	2.18 (2.00, 2.35)	2.29 (2.19, 2.40)	-0.11 (-0.30, 0.07)	0.21
SBP	122.41 (120.07, 124.74)	127.78 (126.47, 129.08)	-5.37 (-7.85, -2.88)	0.0002
DBP	82.71 (80.80, 84.62)	88.07 (86.63, 89.30)	-5.35 (-7.46, -3.24)	<0.0001
RHR	78.43 (76.96, 79.90)	80.21 (78.22, 82.19)	-1.77 (-4.06, 0.51)	0.12
SpO_2	98.15 (98.02, 98.28)	98.02 (97.89, 98.14)	0.13 (-0.03, 0.29)	0.10

Values are mean, 95% CI. SBP: systolic blood pressure, DBP: diastolic blood pressure. RHR: resting heart rate, SpO_2: oxygen saturation. Differences between treatments were assessed by the Mann-Whitney test.

Table S5. Analysis of Δ Neuropeptides and Δ Circadian rhythm biomarkers in cherry and placebo groups.

**Variable**	**Treatment**		**Difference (Cherry-Placebo)**	
	**Cherry (n=19)**	**Placebo (n=21)**	**Mean, 95% CI**	**p value**
Neuropeptides				
**Δ Neurotensin**	16.60 (0.12, 33.08)<br>n = 18	23.87 (8.61, 39.12)	-7.26 (-29.72, 15.19)	*0.60*
**Δ Substance P**	6.71 (0.14, 13.29)<br>n = 18	6.19 (0.10, 12.28)	0.52 (-8.43, 9.48)	0.90
**Δ Oxytocin**	29.35 (-9.73, 68.44)<br>n = 18	24.69 (-11.49, 60.87)	4.66 (-48.60, 57.93)	*0.84*
Circadian Rhythm				
**Δ Cortisol**	-3.24 (-113.03, 106.55)<br>n = 18	60.79 (-40.85, 162.44)	-64.04 (-213.66, 85.58)	0.39
**Δ Melatonin**	1.61 (-5.00, 8.22)<br>n = 18	11.03 (4.22, 17.83)<br>n = 17	-9.41 (-18.90, 0.07)	0.06

Values are mean, 95% CI. Differences between cherry and placebo groups were analyzed by the Mann-Whitney test or by the unpaired t test. Δ: D30-D1. Italicized p values are for log transformed data; untransformed data are presented.

Table S6. Spearman correlation analysis in cherry and placebo groups

**Variables**	**Cherry**							
	**Δ *Bilophila***	**Δ *A. hadrus***	**Δ *R. intestinalis***	**Δ *A. shahii***	**Δ *Bifidobacterium***	**Δ SBP**	**Δ DBP**	**Δ IFNγ**
**Δ DSF**	r = -0.37 p = 0.11	r = -0.09 p = 0.70	r = 0.30 p = 0.27	r = -0.16 p = 0.59	r = 0.15 p = 0.54	r = -0.01 p = 0.95	r = -0.02, p = 0.99	r = 0.07 p = 0.77
**Δ DSB**	r = -0.24 p = 0.30	r = 0.07 p = 0.76	r = 0.26 p = 0.33	r = -0.34 p = 0.27	r = 0.05 p = 0.81	r = -0.39 p = 0.09	r = -0.19, p = 0.42	r = 0.05 p = 0.82
	**Placebo**							
**Variables**	**Δ *Bilophila***	**Δ *A. hadrus***	**Δ *R. intestinalis***	**Δ *A. shahii***	**Δ *Bifidobacterium***	**Δ SBP**	**Δ DBP**	**Δ IFNγ**
**Δ Neurotensin**	r = -0.03 p = 0.90	r = 0.43 p = 0.04	r = 0.58 p = 0.08	r = 0.28 p = 0.34	r = 0.12 p = 0.60	r = -0.01 p = 0.97	r = -0.23 p = 0.31	r = -0.13 p = 0.60
**Δ Melatonin**	r = 0.09 p = 0.71	r = -0.15 p = 0.50	r = 0.46 p = 0.17	r = 0.04 p = 0.88	r = -0.32 p = 0.17	r = -0.21 p = 0.34	r = -0.04 p = 0.85	r = 0.54 p = 0.02
