# Supplementary figures and images for: Assessing the Role of Dark Sweet Cherry (Prunus avium L.) Consumption on Cognitive Function, Neuropeptides, and Circadian Rhythm in Obesity: Results from a Randomized Controlled Trial
Source: Nutrients. 2025 Feb 24;17(5):784. doi: 10.3390/nu17050784 (PMC11901987; doi:10.3390/nu17050784)

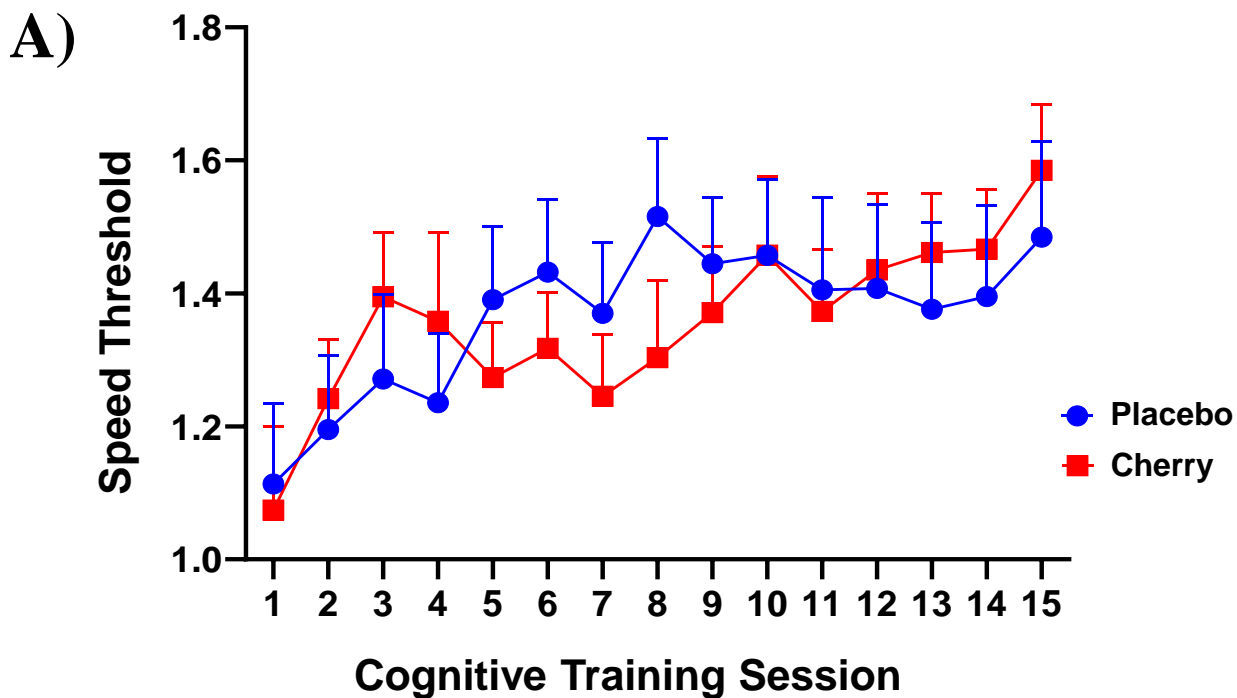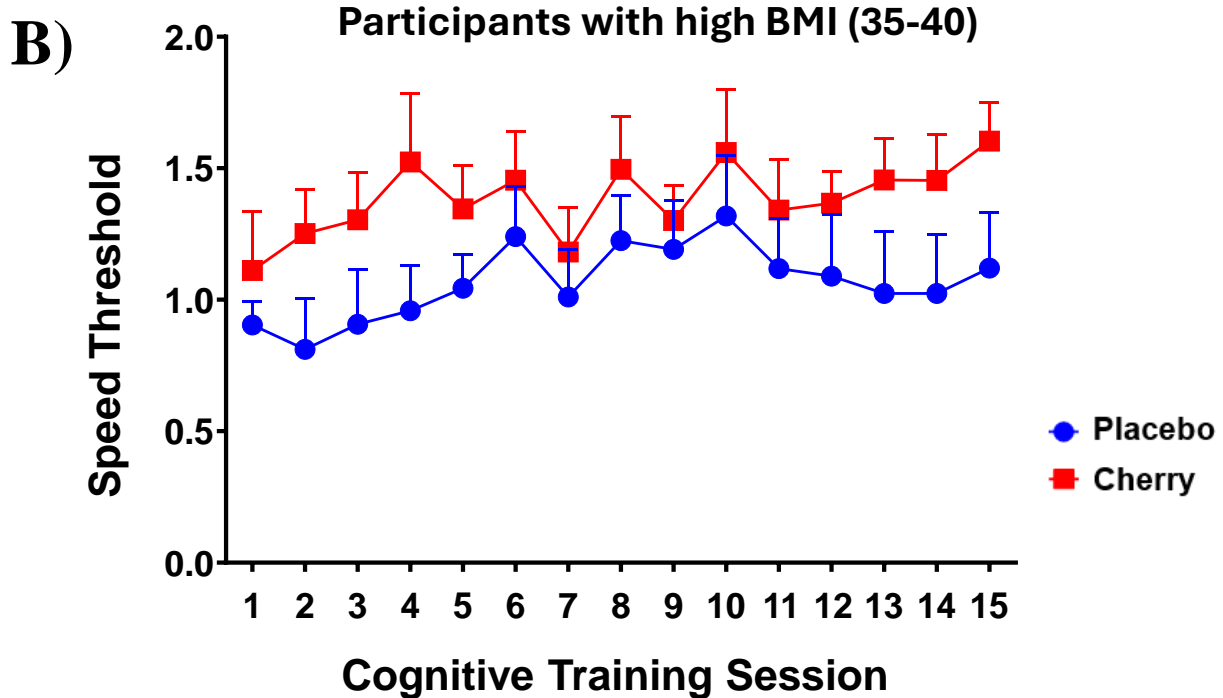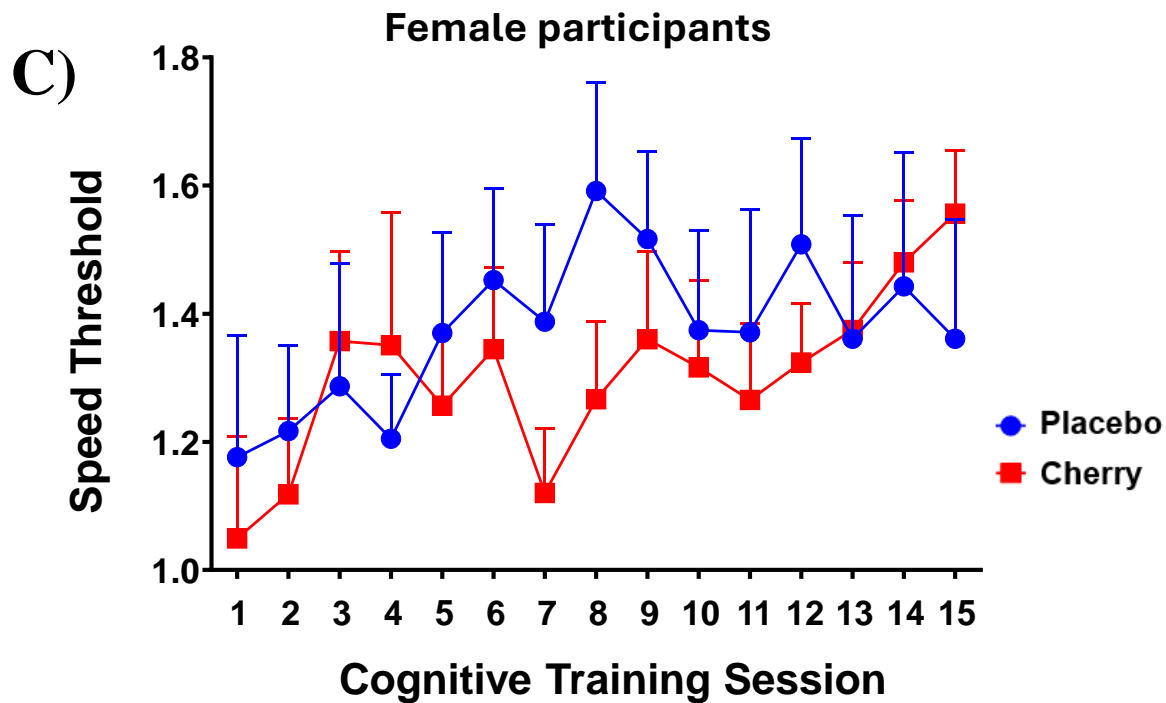

Supplement: Supplementary file 1 [file nutrients-17-00784-s001.zip › Supplementary Figure S1.pdf]
